# A Systematic Review on the Use of Wearable Body Sensors for Health Monitoring: A Qualitative Synthesis

**DOI:** 10.3390/s20051502

**Published:** 2020-03-09

**Authors:** Annica Kristoffersson, Maria Lindén

**Affiliations:** School of Innovation, Design and Engineering, Mälardalen University, 722 20 Västerås, Sweden; maria.linden@mdh.se

**Keywords:** health monitoring, IoT, physical activity monitoring, qualitative synthesis, remote health management, research shortcomings, sensor systems, user demography, wearable body sensors

## Abstract

The use of wearable body sensors for health monitoring is a quickly growing field with the potential of offering a reliable means for clinical and remote health management. This includes both real-time monitoring and health trend monitoring with the aim to detect/predict health deterioration and also to act as a prevention tool. The aim of this systematic review was to provide a qualitative synthesis of studies using wearable body sensors for health monitoring. The synthesis and analysis have pointed out a number of shortcomings in prior research. Major shortcomings are demonstrated by the majority of the studies adopting an observational research design, too small sample sizes, poorly presented, and/or non-representative participant demographics (i.e., age, gender, patient/healthy). These aspects need to be considered in future research work.

## 1. Introduction

The use of wearable body sensors for health monitoring as a means for supporting clinical and remote health monitoring in real-time and to provide health trend monitoring with the aim to predict/prevent health deterioration has the potential to lower the burden on the healthcare system and thereby reduce healthcare costs. The number of available wearable and wireless body sensors and systems are rapidly growing. Simultaneously, research on more energy-efficient and more accessible/smaller sensors for acquiring data as well as research on automatic data analysis of the Big Data, which the sensor-based systems are expected to generate, is being conducted. This advanced data analysis has the potential of generating personalized diagnoses and providing recommendations on treatments at a personalized level. While a promising area, we argue that the data collected for generating advanced data analysis algorithms need to come from participants representing the expected users of these systems.

This systematic review provides a qualitative synthesis of the articles retrieved on using wearable body sensors for health monitoring. We analyze the articles from many perspectives including author affiliations in countries, publication years, context of use, sensor category, research methodology, sample sizes, and participant demographics (i.e., age, gender, patient/healthy). This analysis has identified a number of shortcomings in prior research with respect to both sample size, but also to participant demographics where the latter strongly affects the validity of the results. These shortcomings need to be considered in future research, not only for understanding the user experience, but also to ensure that the advanced data analysis algorithms can reason on data which are representative and valid for the expected users of the systems.

## 2. Methodology

Following the requirements of MDPI Sensors, a systematic review following the PRISMA guidelines [1] was conducted. A total of seven databases were searched, including: Web of Science Core Collection, MEDLINE, Scopus, ScienceDirect, Academic Search Elite, ACM Digital Library, and IEEE Xplore.

The searches were conducted on 24–25 April 2019. The search phrases resulting in the identification, and addition to an EndNote database, of related articles are shown in Table 1. During the search, the keywords were changed in order to broaden or narrow the number of articles found using the previous search phrase. For example, “Ecare” or “mHealth” or “ehealth” was replaced with “care” or “Health” in the second search in Web of Science Core Collection. The same search phrase was used for MEDLINE but it resulted in thousands of hits in SCOPUS. Several additional searches aiming at limiting the number of hits were conducted resulting in "care" or "Health" being replaced with the original search phrase "ecare or mhealth or ehealth" and the exclusion of "feedback" and "pilot application". The search phrase used for Scopus resulted in no hits in Science Direct. Therefore, two less narrow searches were conducted. Variations of these phrases were used in Academic Search Elite, ACM Digital Library and IEEE Xplore.

### Article Selection, Inclusion and Exclusion Criteria

The search resulted in 495 articles. Thereafter, the articles were screened in several steps using EndNote:. Thirty duplicated articles were eliminated and 288 articles were excluded after reviewing each title and abstract individually. Abstracts and articles retrieved that did not match the main research question were excluded from further consideration. For example, we excluded articles on studies using solely environmental exposure sensors or smart home sensors.

Then, pdf copies of all remaining articles were downloaded. Copies of abstracts, introductions and conclusions were extracted to OneNote after which an additional screening was conducted. The eligibility criteria for inclusion in the review were:

- Articles should be published as a journal article or in conference proceedings.
- Articles should consider wearable technology and monitoring.
- Articles should present results from studies where sensor data were collected using humans. Alternatively, the articles present information on a system where the user trial is planned for but not conducted yet.
- Articles should be in English.

**Figure 1:**
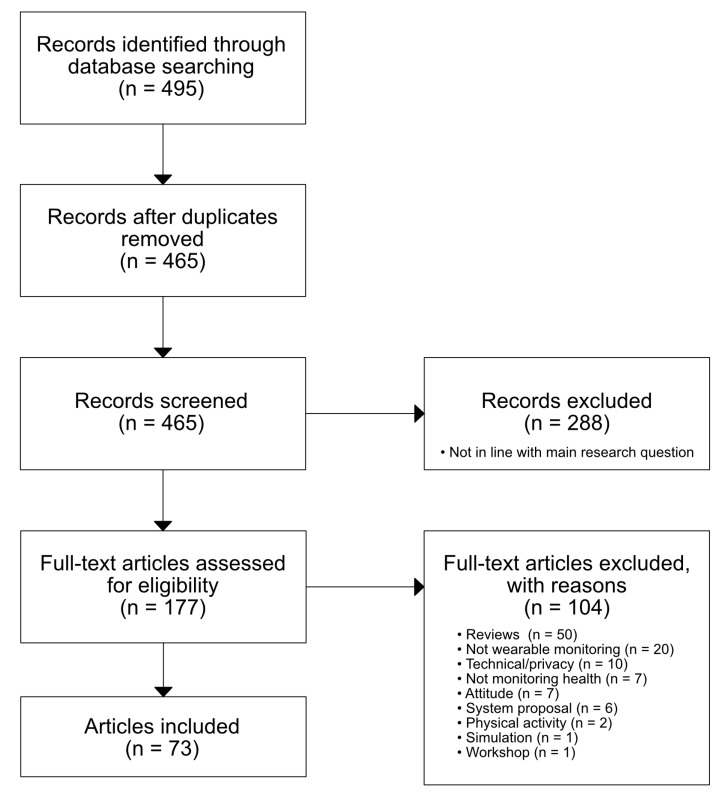
The article selection process.

Overviewing the remaining 177 articles, it was found that the number of publications relating to some health conditions, henceforth called article categories, was low. Therefore, no articles were excluded based on publication year. In addition, we excluded the numerous review articles from further analysis as they cannot be considered original research, i.e., the review articles retrieved were excluded since they do not directly report on a conducted study of people or on the planning of such a study. Publications that met the inclusion criteria, and therefore, considered for further reviewing were 73. The study selection process is depicted in Figure 1.

## 3. Qualitative Synthesis

**Figure 2:**
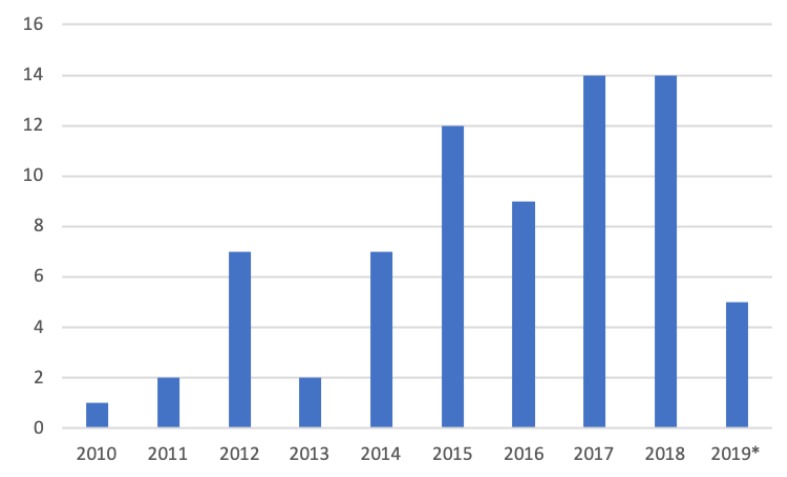
Number of articles per year. * only the articles published prior to 24 April 2019 are counted.

**Figure 3:**
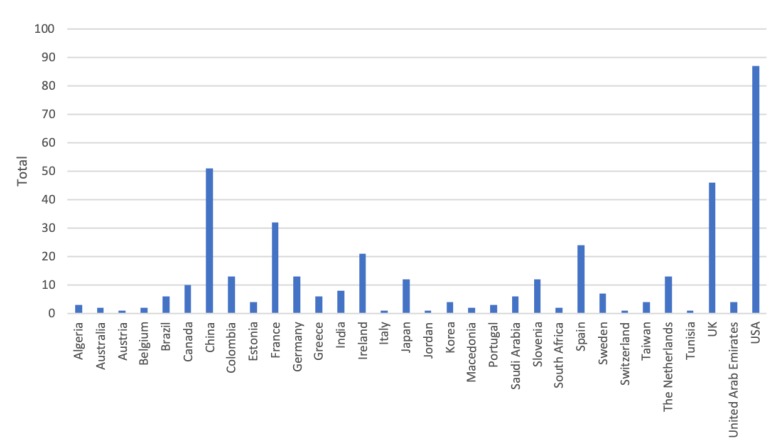
Number of authors affiliated in each country. Authors are calculated for each article, i.e., an author may be calculated more than once and in more than one country.

**Figure 4:**
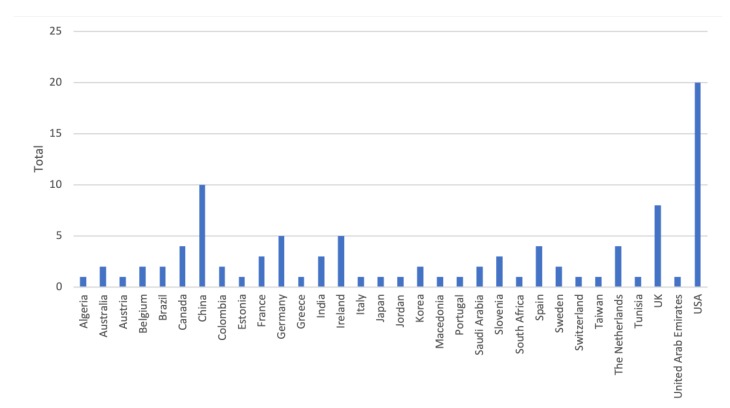
Number of articles per country. Papers with several authors may be counted for several countries.

**Figure 5:**
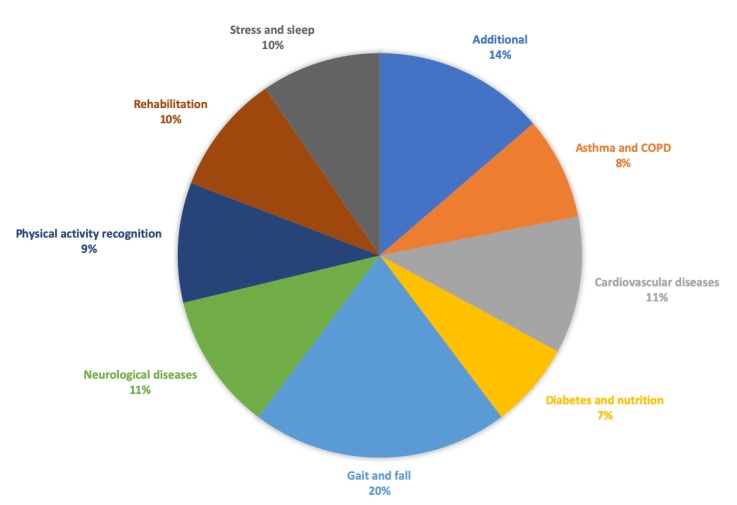
Category-wise distribution of the selected articles. Number of articles for Additional = 10, Asthma/COPD = 6, Cardiovascular diseases = 8, Diabetes and nutrition = 5, Gait and fall = 15, Neurological diseases = 8, Physical activity recognition = 7, Rehabilitation = 7, Stress and sleep = 7.

Inspired by Kekade et al.’s review from 2018 [2], we conducted a qualitative synthesis of the 73 included research articles. They were published between 2010 and 2019, i.e., spanning approx. 9.5 years, among which one article was published in 2010, two in 2011, seven in 2012, two in 2013, seven in 2014, twelve in 2015, nine in 2016, fourteen in 2017, fourteen in 2018 and five before April 24th 2019, see Figure 2. In average, 7.6 articles were published per year during the period 2010–2018. The authors of the 73 research articles were affiliated in 32 countries representing six continents (Africa, Asia, Australia, Europe, North America and South America). See Figure 3 and Figure 4 for further information on which countries authors are affiliated in and the number of publications per country with affiliated authors. The articles were sorted into the following article categories: Asthma/COPD, Cardiovascular diseases, Diabetes and nutrition, Gait and fall, Neurological diseases, Physical activity recognition, Rehabilitation, and Stress and sleep. All articles not directly related to any of the aforementioned article categories were sorted into an article category named Additional. Figure 5 depicts the category-wise distribution of the selected articles. Studying the distribution of articles related to health and physical activity monitoring respectively, it can be seen that 47 % of the articles were related to health (Asthma/COPD, Cardiovascular diseases, Diabetes and Nutrition, Neurological diseases, and Stress and sleep). As much as 39 % of the articles were related to physical activity monitoring (Gait and fall, Physical activity recognition, and Rehabilitation). It is unclear why such a large portion of the articles were related to physical activity monitoring. Possible reasons include that it is easier to monitor physical activity using sensors whereas measures relating to health, e.g., vital signs, need to be provided in a more timely manner.

Sixty research articles reported on studies conducted with people at some level, these are reported in Table 2. We categorized the sensors according to the sensor categories used in [2], namely, physical activity, vital signs, electrocardiography (ECG) and other. Studies reporting on devices measuring movement or activity were classified under the sensor category physical activity. Vital signs include the parameters: blood pressure (BP), body temperature (BT), respiratory rate (RR), heart rate (HR)/pulse, and peripheral oxygen saturation (SpO_2_). Studies measuring ECG were classified under ECG. Finally, studies using sensors for diabetes, swallowing, etc., or a combination of sensors from several sensor categories were classified under the sensor category other. The sensor categories physical activity and other include 23 studies each, vital signs includes three studies, and ECG includes ten studies reported upon in seven research articles.

Similarly to Kekade et al. 2018 [2], we also assessed the studies’ reporting of research design (Table 2), and the reported participant demography, i.e., number of participants, age, gender and the distribution of healthy participants and patients (see Section 3.1, Section 3.2, Section 3.3 and Section 3.4 and Table 3). Many studies presented the participant demographics poorly, or not at all [3,4,5,6,7,8,9,10,11,12]. Rather than excluding these from the tables, we indicate missing information with a “-”. However, we question the fact that all these studies were accepted for publication without providing any information on the participants. Our findings are further discussed in Section 4.

For completeness, the remaining 13 articles not listed in Table 2 and Table 3 were distributed over eight article categories: Asthma/COPD [63], Cardiovascular diseases [64], Gait and fall [65,66,67], Neurological diseases [68], Physical activity recognition [69], Rehabilitation [70], Stress and sleep [71], and Additional [72,73,74,75]. Six articles report on systems where studies are upcoming [63,64,72,73,74,75]. One of them [64] is a continuation of the study reported in [23]. Three articles report on studies using datasets [66,67,69]. Two articles report on qualitative studies of observational and/or interview nature [68,70]. The continuation of the qualitative study [70] is reported upon in [48]. The evaluation in [65] is not clearly presented and the system developed in [71] uses wearable body sensors only to collect ground truth data for a contactless sleep monitoring system. Therefore, [71] was excluded from further qualitative analysis.

### 3.1. Research Methodology

Table 2 reports on the four research designs identified while analyzing the research articles: case-control, crossover, randomized control and observational. Articles categorized as adopting a case-control research design are prospective and include studies with two groups. In most articles, one group is a healthy control group and the other a group sharing an illness. However, in this review, also articles comparing the measures for two distinct groups (e.g., non-shift workers in rural and urban areas) have been categorized as adopting a case-control research design. Articles categorized as adopting a randomized-control research design have participants with the same background being randomly assigned to one of two study conditions. One article has been categorized as a crossover study [59], the participants have experienced both study conditions but in randomized order. The articles categorized as being observational are typically conducted in a controlled fashion during which data are collected. In this review, the majority of the articles were categorized as being observational. A few articles adopted a case-control [19,41,42,50,54] or randomized control research design [16,28,56]. For some articles [11,18,21,23,24,58], information provided on how the experiments were conducted was not sufficient for determining the research design adopted.

**Figure 6:**
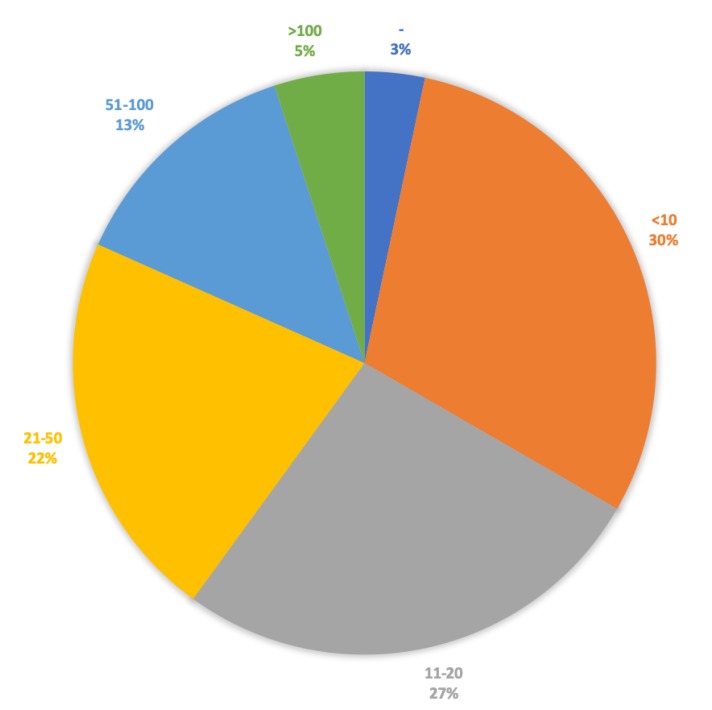
Distribution of the number of participants per included study. - denotes studies which did not provide information on number of participants.

Studying the number of participants included in the studies, we first summarized the number of participants in the cases where an article reported on several smaller studies. It can be seen from Figure 6 that 57% of the studies were conducted with up to 20 participants and that 30% were conduced with 10 or fewer participants. Only 40% of the studies were conducted with 21 or more participants (22% collected 21-50 participants, 13% had 51–100 participants leaving 5% with more than 100 participants).

**Figure 7:**
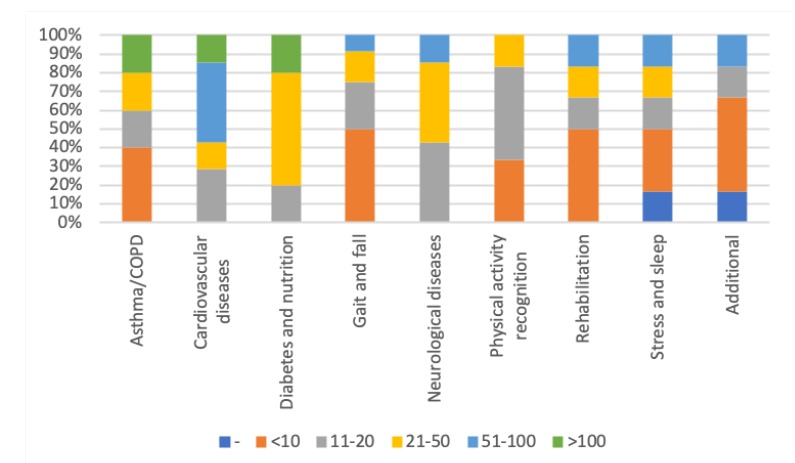
Distribution of the number of participants per article category. - denotes studies which did not provide information on number of participants.

Looking more closely into each article category, Figure 7 shows that the majority of the studies within the categories Asthma/COPD, Gait and fall, Physical activity recognition, Rehabilitation, Stress and sleep, and Additional were conducted with up to 20 participants. The studies with more than 100 participants fall within the categories Asthma/COPD, Cardiovascular diseases, and Diabetes and nutrition. Studies with 51–100 participants were conducted within the categories Cardiovascular diseases, Gait and fall, Neurological diseases, Rehabilitation, Stress and sleep, and Additional.

To make technical validations that a sensor is working, a small number of participants can be accepted. However, to be used in clinical investigations, power calculations taking the research question into account should be used to decide the number of needed participants.

### 3.2. Age Distribution

Information on the participants’ age was provided in 35/60 (58.3%) of the articles reporting on data collection studies with people (Table 3). Another two articles [21,23] provided the information on age for only one of the study groups. A very limited number of studies were conducted with people where $\mu_{age}$ > 65 [40,53] or $\mu_{age}$ > 60 [13,14,48,51]. Two studies [30,39] were conducted with one young group and one group where $\mu_{age}$ > 65, whereas $\mu_{age}$ > 60 for one of the groups in [17]. Two articles report on studies with large age ranges where some participants exceed 65 years of age (16–72 and 20–73 in [22], and 40–70 in [35]).

Studying the articles from an article category perspective, none of the studies reporting on the categories Cardiovascular diseases, Diabetes and nutrition, Other or Stress and sleep was conducted with participants where $\mu_{age}$ > 60. The categories Asthma/COPD, Gait and fall, Neurological diseases, and Rehabilitation include some studies with this age group. None of the studies within the Physical activity recognition category report on the participants’ age.

### 3.3. Gender Distribution

Information on the participants’ gender was provided in 33/60 (55%) of the articles reporting on data collection studies with people (Table 3). Three more articles [9,13,23] reported on studies with more than one group but not the gender for all groups.

Studying the articles from an article category perspective, all Asthma/COPD studies except [13] provided full information on gender distribution. The latter, [13] also reports on a study with a subset of the participants without providing information on gender. Regarding cardiovascular diseases studies, only [20,23] provided full information on gender distribution. Another 20 want to participate in screening although the study described in [23] is not approved yet by an ethical committee. All but one study within Diabetes and nutrition report on gender. The majority of the studies within Gait and fall contain information on gender. More than half (57%) of the articles on Neurological diseases and 50% of the articles on Other present information on gender. Regarding the category Physical activity recognition, only one article [47] provides full information on gender. Another article, [9] provides information on gender for one of their four sub-studies. The majority (80%) of the Rehabilitation studies and 50% of the Stress and Sleep studies provide gender information.

Studying the articles from a gender distribution perspective, the vast majority of the participants in the studies reporting on Asthma/COPD are men. For Cardiovascular diseases, [20] had a rather even gender distribution, [23] reported on gender in a study aiming at validating a measurement protocol and for evaluating the usability and acceptance level of an ICT equipment. The majority of the participants were men. A similar pattern is observed for Diabetes and nutrition, Gait and fall, Neurological diseases, Other, Rehabilitation and Stress and sleep. Women are only in majority for one of the groups in the Gait and fall study [30], and the Rehabilitation studies [41,42,48].

### 3.4. Tests on Patients and Healthy Users

Information on whether the participants were patients and/or healthy was provided in 39/60 (65%) of the articles (Table 3). An additional four studies, [9,21,22,38] present the distribution of patients and/or healthy for some of the reported sub-studies. Two groups including 84 participants in total were representing patients and healthy participants in [19]. Seven articles [13,17,19,23,41,42,50] report on the conduction of studies with both patients and healthy. Two articles [9,22] contain results from several sub-studies and while not providing patient/healthy information for all sub-studies, claim to have used both patients and healthy participants during data collection. For several article categories, many of the studies reported information on both patients and healthy users.

Studying the articles from a health perspective, i.e., looking particularly at the article categories Asthma/COPD, Cardiovascular diseases, Diabetes and nutrition, Neurological diseases, and Stress and Sleep, the reporting and/or use of patients/healthy participants varies. Almost all participants in studies on Asthma/COPD and Neurological diseases were patients. Surprisingly, the Cardiovascular diseases [20,23] were conducted solely with healthy participants while another [21] and three of the sub-studies in [21,22] lack information on whether the participants were healthy or patients. Regarding Diabetes and Nutrition, two works [24,28] were conducted with patients, one study [27] was conducted with healthy participants while two articles [25,26] lack this information. Finally, regarding Stress and sleep, none of the studies report on studies with patients. Three articles [55,57,58] were conducted with healthy participants while the remaining three articles lack this information.

Studying the articles from a physical activity perspective, i.e., looking particularly at the article categories Gait and fall, Physical activity monitoring and Rehabilitation. No information on whether the participants were healthy or patients were provided in the articles falling under the Physical activity monitoring article category. None of the studies within Gait and fall used patients. The picture is mixed for the category Rehabilitation, two studies were conducted solely with patients [48,51] whereas [50] reports on two sub-studies conducted with patients and healthy participants respectively. One work [52] was conducted solely with healthy participants and two works [49,53] do not provide this information.

## 4. Discussion and Conclusions

In this systematic review, we provide a qualitative synthesis on retrieved articles on using wearable body sensors for health monitoring. The articles found were categorized as relating to: Asthma/COPD, Cardiovascular diseases, Diabetes and Nutrition, Gait and fall, Neurological diseases, Physical activity recognition, Rehabilitation, Stress and sleep, and Additional. Section 3 provided a qualitative synthesis of the studies with respect to research methodology and participant demography, i.e., number of participants, age, gender and the distribution of healthy participants and patients. Using this information, we have identified a number of shortcomings. Below follows a discussion on these shortcomings in relation to prior research.

There are many age-related health issues such as changing biological factors, the onset of illnesses which are often chronic and the decline of cognitive abilities. For example, “fall prediction is a challenging problem due to the combination of intrinsic and extrinsic fall risk factors that contribute to a fall. Intrinsic factors include age, fall history, mobility impairments, sleep disturbances, and neurological disorders", pp. 1 [76]. It is reported in [77] that 35% of non-institutionalized adults had abnormal gait and that sleep disturbances are very common among older people. Further, chronic conditions affect physical activity levels, and activities such as rising from a chair is demanding for older people [77]. It is clear that the whole motion pattern changes with age and the onset of illnesses related to the human locomotor system. Yet, the majority of the studies focusing on gait and fall in this review were simulations that include none or few old participants. This shortcoming is also discussed in [76], “It is evident that existing systems have mainly been tested in laboratory environments with controlled conditions and do not include frequent fallers and aging adults as test subjects.[..] future work should focus on longitudinal studies of fall detection and prediction systems in real-life conditions on a diverse group that includes frequent fallers, aging adults, and persons with neurological disorders.” p.8 [76]. Not studying the sensor systems in real-life conditions affect the validity of the results since the performance is not studied in realistic conditions. The low number of studies with older people is also a shortcoming since age-related issues are not taken into consideration to a sufficient degree.

There are many differences between the two genders. As a first example, we want to mention the American Heart Association’s (AHA) scientific statement from 2016 [78] on acute myocardial infarction (AMI) in women. “Sex differences occur in the pathophysiology and clinical presentation of MI and affect treatment delays.”, p. 932 [78]. Further, AHA reports that the same perfusion therapies are recommended despite the fact that the risk of bleeding or other complications is higher among women. Further, women are being under-treated with guideline recommendations. This results in increased readmission, re-infarction, and death rates during the first year after a myocardial infarction. Cardiac rehabilitation is also underused and under-prescribed among women [78]. On the same lines, the results of a cohort study [79] with almost 5000 patients $\mu_{age}$ > 65 who were admitted to 366 US hospitals in the period 2003–2009, has found that women are less likely to receive optimal care at discharge. Yet, only two of the studies retrieved within the category Cardiovascular diseases provide information on the participants’ gender. This is not the only shortcoming for studies on Cardiovascular diseases however. Several studies, or sub-studies, were conducted with very large age spans without the provision of a mean age. Others were conducted with young people or lacks information on age. Further, several works report on studies with healthy participants.

Hence, studies taking both genders into consideration, but also the age factor, are highly desired in the category Cardiovascular diseases. Not including information on gender and/or not considering gender/sex during data collection is a shortcoming regardless of the category to which a study belongs. It is argued in [80] that there are areas were specific data on women is lacking while specific data on men is missing in other areas.

Regitz-Zagrosek [80] outlines a number of differences between men and women. These include: women more frequently having anemia, women suffering from coronary artery disease in average ten years later than men, a higher frequency of boys having asthma in young ages while the frequency changes to young adulthood, diabetes increasing the risk for coronary heart disease more among women, and osteoporosis being more frequent in women but under-diagnosed in men. Osteoporosis disease is characterized by a decreased bone mass density and a disrupted normal trabecular architecture reducing bone strength [81]. Therefore, Osteoporosis increases the risk of fractures after a fall but no symptoms of the disease are shown until a fracture occurs [80]. According to [81], there are several factors relating to Osteoporosis which increases the risk of falling. These include the fear of falling, which increases the risk of falling [82,83]. In addition, [81] reports on studies discussing women with osteoporosis or low bone mass where fear of falling is associated with more falls [84], and the confidence in balance is related to balance and mobility [85]. Further, [84] reports that an increased thoracic kyphosis is associated with recent falls among women with Osteopororosis. I.e., women with thoracic kyphosis were more likely to have had a recent fall. Thoracic kyphosis is an abnormal convex curvature of the spine at chest height which is much more common among older women than men due to estrogen losses [86]. All these works [81,82,83,84,85] date from 2004-2011, hence it is astonishing that some articles retrieved within the article category Gait and fall have not reported information on gender and that some other articles were conducted solely with men. Hence, we argue that future studies in the categories discussed in this article must take gender into consideration. This shortcoming was also highlighted in [2].

Undoubtedly, healthy participants and patients differ in many aspects. Yet, only 65% of the studies overall reported this information. A positive example here is the fact that the studies reported upon in the category Asthma/COPD were conducted almost entirely with patients. This indicates that the results in this area are reliable. On the contrary, none of the studies within Gait and fall, or Stress and sleep have reported that the studies were conducted with patients. Also [76,77] have previously discussed the shortcoming of not conducting studies with patients in the category Gait and fall. Considering the research question for this review article, we question the fact that 35% of the retrieved articles lack information on whether the participants were healthy or patients. We argue that the use of healthy participants, or not providing this information, affect the validity of the study results. Future studies need to consider the inclusion of patients to a further extent.

Studying the sample size in the reported studies, 56% of the articles report on studies conducted with up to 20 participants, and only 20% of the articles report on studies conducted with 51 or more participants. The distribution of numbers vary between categories. The majority of the studies reported in the categories Asthma/COPD, Gait and fall, Physical activity recognition, Rehabilitation, and Stress and sleep were conducted with up to 20 participants. We find the overall low number of participants a shortcoming and recommend that future studies are conducted with larger study samples. However, taking demographic factors, i.e., age, gender and healthy/patient into consideration is highly needed prior to increasing the sample sizes in studies on health monitoring using wearable body sensors.

## Figures and Tables

**Table 1:** An overview of search phrases and databases used during article retrieval.

Database	Search Phrase	Number of Articles
Web of Science Core Collection	ALL FIELDS: ((“body sensor" or “wireless body sensor” or “wireless wearable technology” or “biomedical sensor” or “IoT”) and (“Ecare” or “mHealth” or “eHealth’) and (“Social impact” or “Compliance” or “Acceptance” or “Clinical trial” or ‘Pilot test” or ‘Human input” or “Feedback” or “Pilot application” or “Human in the loop”))	7
Web of Science Core Collection	ALL FIELDS:((“body sensor” or “wireless body sensor” or “wireless wearable technology” or “biomedical sensor" or “IoT”) and (“care" or “Health”) and (“Social impact” or “Compliance” or “Acceptance” or “Clinical trial" or “Pilot test” or “Human input” or “Feedback” or “Pilot application" or “Human in the loop”))	142
MEDLINE (Web of Science)	TOPIC: ((((((“body sensor”) OR “wireless body sensor”) OR “wireless wearable technology”) OR “biomedical sensor”) OR “IoT”) AND (“care”) OR “Health”)) AND ((((((((“Social impact”) OR “Compliance”) OR “Acceptance”) OR “Clinical trial”) OR “Pilot test”) OR “Human input”) OR “Feedback”) OR “Pilot application") OR “Human in the loop")) Timespan: All years. Indexes: MEDLINE.	25
Scopus	ALL(body sensor OR wireless body sensor OR wireless wearable technology OR biomedical sensor) AND (ecare OR mhealth OR ehealth) AND ( Social impact OR compliance OR acceptance OR Clinical trial OR Pilot test ) Limiting to English	187
ScienceDirect	Title, abstract, keywords: “wearable sensors” and health and impact. Limited to review articles, research articles, conference abstracts, case reports.	13
ScienceDirect	Title, abstract, keywords: “body sensor” and health and impact. Limited to review articles, research articles, conference abstracts, case reports.	5
Academic Search Elite	Free text search: “body sensor” and health and impact English.	8
Academic Search Elite	Free text search: “body sensor” and health and acceptance	3
ACM Digital Library	(+“body sensor” +and +health +and +impact)	12
IEEE Xplore	“body sensor” and health and impact	81
IEEE Xplore	“body sensor” and health and trial	12

**Table 2:** List of articles reporting on conducted studies. —indicates that information is missing.

Author, Year	Ref.	Article Category	Research Design	No. of Participants	Sensor Category
Bonnevie et al. 2019	[13]	Asthma/COPD	Observational	104	Vital signs
				5	
Caulfield et al. 2014	[14]	Asthma/COPD	Observational	10	Physical activity
Estrada et al. 2016	[15]	Asthma/COPD	Observational	1	Other
Katsaras et al. 2011	[16]	Asthma/COPD	Randomized control	48	Other
Naranjo-Hernández et al. 2018	[17]	Asthma/COPD	Observational	2	Vital signs
				9	
Huang et al. 2014a	[18]	Cardiovascular diseases	-	225	ECG
Huang et al. 2014b	[19]	Cardiovascular diseases	Case-control	84	ECG
Javaid et al. 2018	[20]	Cardiovascular diseases	Observational	60	Other
Li et al. 2019	[3]	Cardiovascular diseases	Observational	16	Other
Raad et al. 2015	[21]	Cardiovascular diseases	-	30	ECG
			-	2	
Simjanoska et al. 2018	[22]	Cardiovascular diseases	Observational	16	ECG
				3	
				25	
				7	Dataset ECG
Susič and Stanič 2016	[23]	Cardiovascular diseases	-	13	ECG
Al-Taee et al. 2015	[24]	Diabetes and nutrition	-	22	Other
Alshurafa et al. 2014 and Alshurafa et al. 2015	[25,26]	Diabetes and nutrition	Observational	10	Other
				20	
Dong and Biswas 2017	[27]	Diabetes and nutrition	Observational	14	Other
Onoue et al. 2017	[28]	Diabetes and nutrition	Randomized control	101	Physical activity
Atallah 2012	[29]	Gait and fall	Observational	34	Physical activity
Godfrey et al. 2014	[30]	Gait and fall	Observational	24	Physical activity
Lee et al. 2015	[31]	Gait and fall	Observational	11	Physical activity
Liang et al. 2012	[32]	Gait and fall	Observational	8	Physical activity
Liang et al. 2018	[33]	Gait and fall	Observational	18	Physical activity
Paiman et al. 2016	[34]	Gait and fall	Observational	2	Other
Tino et al. 2011	[35]	Gait and fall	Observational	3	Other
Williams et al. 2015	[36]	Gait and fall	Observational	5–6	Physical activity
Wu et al. 2013	[4]	Gait and fall	Observational	7	Physical activity
Wu et al. 2019	[37]	Gait and fall	Observational	15	Physical activity
Zhao et al. 2012	[38]	Gait and fall	Observational	8	Physical activity
Zhong et al. 2019	[39]	Gait and fall	Observational	56	Physical activity
Giuberti et al. 2015	[40]	Neurological diseases	Observational	24	Physical activity
Gong et al. 2015, Gong et al. 2016	[41,42]	Neurological diseases	Case-control	41	Physical activity
Kuusik et al. 2018	[43]	Neurological diseases	Observational	51	Physical activity
Sok et al. 2018	[44]	Neurological diseases	Observational	13	Physical activity
Stamate et al. 2017 and Stamate et al. 2018	[45,46]	Neurological diseases	Observational	12	Other
Castro et al. 2017 and Rodriguez et al. 2017	[5,6]	Physical activity recognition	Observational	3	Other
Doron et al. 2013	[7]	Physical activity recognition	Observational	65	Other
				20	
Rednic et al. 2012	[47]	Physical activity recognition	Observational	17	Physical activity
Xu et al. 2014	[8]	Physical activity recognition	Observational	14	Other
Xu et al. 2016	[9]	Physical activity recognition	Observational	4	Other
				3	Physical activity
				5	
				6	
Argent et al. 2019	[48]	Rehabilitation	Observational	15	Physical activity
Banos et al. 2015	[49]	Rehabilitation	Observational	10	Other
Lee et al. 2018	[50]	Rehabilitation	Case-control	30	Physical activity
Timmermans et al. 2010	[51]	Rehabilitation	Observational	9	Physical activity
Whelan et al. 2017	[52]	Rehabilitation	Observational	55	Physical activity
Xu et al. 2017	[53]	Rehabilitation	Observational	6	Other
Lin et al. 2012	[54]	Stress and sleep	Case-control	18 (6/12)	Physical activity
Nakamura et al. 2017	[55]	Stress and sleep	Observational	4	Other
Parnandi and Gutierrez-Osuna 2017	[56]	Stress and sleep	Randomized control	25	Other
Uday et al. 2018	[57]	Stress and sleep	Observational	10	Other
Umemura et al. 2017	[58]	Stress and sleep	Case-control	54	Other
Velicu et al. 2016	[10]	Stress and sleep	Observational	-	-
Ayzenberg and Picard 2014	[59]	Additional	Crossover	10	Other
Pagán et al. 2016	[60]	Additional	Observational	2	Other
Rawasdeh et al. 2017	[61]	Additional	Observational	55	ECG
Seeger et al. 2012	[11]	Additional	-	-	Other
Wannenburg and Malekian 2015	[12]	Additional	Observational	4–8	Vital signs
Wu et al. 2018	[62]	Additional	Observational	20	ECG

**Table 3:** Demographic information on conducted studies. - indicates that information is missing.

Ref.	Article Category	No. of Participants	Age Group	Age Statistics	Male	Female	Patient	Healthy
[13]	Asthma/COPD	104	57–70	64	67 (64%)	37 (36%)	104	
		5	50–66	62	-	-	5	
[14]	Asthma/COPD	10		61.5 ± 5.7	5	5	10	
[15]	Asthma/COPD	1	-	-	1			1
[16]	Asthma/COPD	48	-	-	48		48	
[17]	Asthma/COPD	2	36 and 42		2			2
		9	55–76	64±6.6	6	3	9	
[18]	Cardiovascular diseases	225	-	-	-	-	225	
[19]	Cardiovascular diseases	84	-	-	-	-	1 group	1 group
[20]	Cardiovascular diseases	60	-	26.9±6.1	28	32		60
[3]	Cardiovascular diseases	16	-	-	-	-	-	-
[21]	Cardiovascular diseases	30	20–23		-	-	-	-
		2	-	-	-	-	2	
[22]	Cardiovascular diseases	16	16–72	-	-	-	-	-
		3	25–27	-	-	-	-	-
		25	20–73	-	-	-	14	11
		7	20–74	-	-	-		7
[23]	Cardiovascular diseases	13	-	50.6±9	8	5		13
[24]	Diabetes and nutrition	22	-	-	-	-	22	
[25,26]	Diabetes and nutrition	10	20–40		8	2	-	-
		20	20–40		12	8	-	-
[27]	Diabetes and nutrition	14	-	-	9	5		14
[28]	Diabetes and nutrition	101	-	57.1±12.5	56	45	101	
[29]	Gait and fall	34	-	28.22±12.77	21	13		34
[30]	Gait and fall	24 (12/12)	20–40	32.5±4.8	7	5		12
				65.0±8.8	5	7		12
[31]	Gait and fall	11	-	27.6±4.3	11			11
[32]	Gait and fall	8	-	23±3.45	8			8
[33]	Gait and fall	18	-	25±3.24	12	6		18
[34]	Gait and fall	2	28 and 24	-	1	1		2
[35]	Gait and fall	3	40–70	-	-	-	-	-
[36]	Gait and fall	5–6 (1/5)	27	-	1		-	-
			21–36	27	4	1	-	-
[4]	Gait and fall	7	-	-	-	-	-	-
[37]	Gait and fall	15	20–27	-	-	-		15
[38]	Gait and fall	8	-	28.5±4.3	-	-		8
[39]	Gait and fall	56 (28/28)	-	24.6±2.7	14	14		28
			>55	66.1±5.0	18	10		28
[40]	Neurological diseases	24	31–79	65.9±12.3	17	7	24	
[41,42]	Neurological diseases	41 (28/13)	-	40.5±9.4	25%	25%	28	13
			-	39.3±10.3	47%	53%		
[43]	Neurological diseases	51	-	-	-	-	51	
[44]	Neurological diseases	13	22–50	-	9	4	13	
[45,46]	Neurological diseases	12	-	-	-	-	12	
[5,6]	Physical activity recognition	3	-	-	-	-	-	-
[7]	Physical activity recognition	65	-	-	-	-	-	-
		20	-	-	-	-	-	-
[47]	Physical activity recognition	17	-	-	10	7	-	-
[8]	Physical activity recognition	14	-	-	-	-	-	-
[9]	Physical activity recognition	4	-	-	-	-	-	-
		3	-	-	-	-		3
		5	-	-	-	-	5	
		6	-	-	3	3	-	-
[48]	Rehabilitation	15	-	63±8.32	6	9	15	
[49]	Rehabilitation	10	21–37	-	8	2	-	-
[50]	Rehabilitation	20	-	54.4±10.1	-	-	20	
		10		53.8±11.4	-	-		10
[51]	Rehabilitation	9	-	60.7	5	4	9	
[52]	Rehabilitation	55	-	24.21±5.25	37	18		55
[53]	Rehabilitation	6	-	72.5±6.0	3	3	-	-
[54]	Stress and sleep	18 (6/12)	19–22 overall	-	5	1	-	-
					11	1	-	-
[55]	Stress and sleep	4	25–36	-	4			4
[56]	Stress and sleep	25	19–33	-	15	10	-	-
[57]	Stress and sleep	10	-	-	-	-		10
[58]	Stress and sleep	54 (26/28)	-	22	-	-		54
			-	21	-	-	-	-
[10]	Stress and sleep	-	-	-	-	-	-	-
[59]	Additional	10	25–35	30.8±4.2	9	1		10
[60]	Additional	2	-	-		2	2	
[61]	Additional	55	18–22	-	50%	50%	-	-
[11]	Additional	-	-	-	-	-	-	-
[12]	Additional	4–8 (4/4)	-	-	-	-	-	-
			-	-	-	-	-	-
[62]	Additional	20	-	-	-	-		20
